# The Social Malady

**Published:** 1914-01

**Authors:** 


### Department of Eugenics.

Conducted by Dr. Malone Duggan and Dr. Theo. Y. Hull.

### THE TEXAS STATE SOCIETY OF SOCIAL HYGIENE.

Headquarters: San Antonio, Texas.

Dr. Malone Duggan, President, San Antonio.
Dr. F. E. Daniel, 1st Vice President, Austin.
Prof. A. Caswell Ellis, 2d Vice President, Austin.
Mrs. Eli Hertzberg, 3d Vice President, San Antonio.
Dr. Theo. Y. Hull, Secretary, San Antonio.
Mr. W. L. Evans, Treasurer, San Antonio.

EXECUTIVE COMMITTEE.

| | | |
|---|---|---|
| Dr. Malone Duggan, Chm. | Hon. J. C. Willacy. | Dr. W. A. King. |
| Dr. Theo. Y. Hull, Sec'y. | Mr. W. L. Evans. | Dr. J. W. Torbett. |
| Dr. W. F. McCaleb. | Dr. Frank D. Boyd. | Dr. F. C. Walsh. |
| Hon. C. H. Jenkins. | Dr. J. R. Nichols. | Dr. Joe Reuss. |
| Rev. A. W. S. Garden. | Dr. J. M. McCutchan. | Dr. Frank Paschal. |

### The Social Malady.

In the last number I discussed those symptoms of the social disorder which have their origin (1) in the living conditions of the people, often finding expression in the high valuation of wealth contrasted with a low valuation of human life,—the antagonism between wealth and poverty; (2) in an excessive morbidity which shortens the average life of one or more decades and levies an enormous tribute from the young and helpless; (3) in an increasing ratio between the abnormal and the normal indicated by the alarming proportion of the mentally deficient and the actually insane, and (4) in that low moral state known as crime.

In this number I wish to discuss those symptoms which have their origin in the conjugal relation of man and woman. It does not require a very high degree of intellectuality to comprehend that the more and more frequent appeals to the divorce courts indicate a growing disregard for the sacredness of marriage—the foundation of the State. Without question marriage is the most important contract into which a human being ever enters. It is potent for the greatest possible good, for on it the whole superstructure of organized society rests. It should be held sacred and inviolate and must be if our social organization is not to disintegrate. Yet thousands upon thousands rush into matrimony without ever a thought of its significance or its binding consequences. A momentary passion, an obliging license clerk and a weakling in whose hands has been entrusted a most important function of the State sums up the chief requirements. They become man and wife in the eyes of the law for a day or a month or possibly a year. An appeal lies to the divorce court, and there begins that long and often disgraceful process of the undoing what the thoughtless have done.

It is said that in this enlightened age not more than one marriage in five is happy. Why should the conjugal yoke be irksome in the other four? A first reason may be found in the unpreparedness of the parties to take the obligation seriously. If any other business in life were held so lightly or entered into with so little of preparation, failure and bankruptcy would almost invariably follow. A second reason may be found in the making of marriage a convenience. A case of this kind came to my notice a few years ago. A man and a woman, each having left their youth behind many summers, met. Each gained the idea that the other had a more or less comfortable income. The woman, a partial invalid encumbered with a dependent relative and possessed of an income hardly sufficient to meet their needs, attended church regularly. The man, also in poor health, needing the comforts of a home, and receiving a salary quite sufficient to meet his needs when he worked, also attended church regularly. They met, were engaged, married. Within a month she discovered she was leaning on a broken reed. In the same time he discovered she was not the nurse he sought. It was a marriage for convenience, but neither found it such. The divorce court, however, was convenient, and it was appealed to with success.

Darwin, when asked if he should discuss the subject of human evolution in his work on the origin of species, replied that it was so surrounded with prejudice that he should avoid it altogether. Any attempt to change our marriage customs invariably meets with opposition. One time when discussing the proposition to further safeguard the marriage relation by requiring a certificate of health as a basis for the issuance of a license, with a group of ministers, one objected because it would interfere with the laws of God. We might ask what is God's intention or purpose in the evolution of the human race. Is it natural selection, the so-called survival of the fittest, or is it the intelligent application of our knowledge to human development? If a child contracts scarlet fever or smallpox is it God's law that the child be at large infecting the community? Sanitary science has taught us better than this. Yet the principle involved in the two questions is precisely the same. The health department does not consider that God has anything to do with the control of contagious diseases, and quarantines the infected person or persons for the public good. We do not consider it breaking God's law to quarantine against yellow fever or cholera. Yet you interfere with the individual's personal liberty no more in the one instance than in the other. It is common sense to restrict the liberty of the sick of a contagious disease, whether the individual is seeking to continue his avocation or seeking a license to marry. We have moved out of that age when man was considered something separate and apart from the rest of creation, when he was thought to be governed by supernatural laws at total variance with those governing the rest of animal nature, and when to hold an autopsy on a human body to discover the cause of death was a crime, for sickness was a dispensation of providence, and a few hundred thousand deaths prematurely was simply increasing the inhabitants of another world for their own good. The many centuries of human experience have taught that the individual suffering with a contagious disease, if unrestrained, is a public menace. When a person suffering with a contagious disease is permitted to marry and inoculates the healthy party, and that party dies from the effects thereof, though a few days only or many years may intervene, it may be God's law, but it is murder just the same.

Yet there are many thousands of such marriages contracted and sanctioned each year, there are thousands of surgical operations that sooner or later follow such marriages, there are other thousands of invalids that owe their condition to this lack of proper supervision, and there are other thousands that die because we seemingly consent to this anomaly in human life, the supposed personal liberty and a feeling that we must not interfere with long established customs, notwithstanding that many customs outlive their usefulness and in time become harmful.

The extent to which prejudice, the slavery to custom and ignorance may be carried is frequently exhibited in our State Legislatures and even in our National Congress when public health legislation is attempted. Public health measures are humanitarian in character and as such are often lacking in support because no selfish purpose is served by their passage. During the last two sessions of the Legislature it has been attempted to introduce bills to safeguard marriage by requiring that the applicants for license be required to furnish certificates from reputable physicians showing freedom from contagious diseases, etc. The first bill, a carefully drawn measure, got as far as the appropriate committee in one branch of the Legislature. Notwithstanding three or four physicians were on the committee, men generally supposed to be familiar with the disastrous consequences of such marriage as the bill sought to prohibit, the measure died of inanition. The second bill was drawn after the law in force in the State of Washington. It was less fortunate than its predecessor. It never even reached the point of being referred to any committee. It was redrawn, some of the most essential features eliminated, the penalty for its violation practically removed, and the whole measure so weakened as to be useless.

So startling are the facts in regard to the abuse of marriage that students of sociology shudder at the consequences that follow and must continue to follow for generations after the evil is corrected. Yet the men who represent the people in our legislative halls obligate themselves to protect the honor and welfare of the

State. With very few exceptions these men have neither the knowledge nor the moral courage to fight for the womanhood and childhood of the State.

This symptom of the social malady is so pronounced that even the blind can see it. Yet it is so entrenched in custom and prejudice that those who are capable of looking ahead, and whose minds and hearts are pure enough to harbor humanitarian ideas are classed as cranks and fanatics. Many a social worker has felt this. Many a minister when he directed his words against the social evil has felt the keen edge of disapproval. The canker worm of customs too long survived, of prejudice, of ignorance, gnaws at the root of the plant called society, while we content ourselves by sprinkling the leaves to cure the blight that fills our hospitals, our orphan asylums, our almshouses, our homes for the feeble-minded, our insane asylums, and our penitentiaries.

We now turn to the fifth symptom of this social malady, which, to the student of social questions, is of the first importance. This symptom is the existence of a decreasing birth rate in that very social class from which every nation must derive its brains and strength. It is not from the highest social ranks that the nation receives its great men, its statesmen, educators, scientists, engineers, financiers, étc. It is not from the lowest strata of society that such men come. It is from this great middle class that the nations have drawn their strength and have depended on the same to maintain the status of society. The appearance of a decreasing birth rate in any class means in time a change in the general character of society, either for the better or worse. So long as the birth rate remains in equilibrium among the various classes, society maintains a fixed condition of mental and moral characteristics. The stability of a people depends upon this condition. The various classes must be in equilibrium. It is a law of nature that a group of organisms will tend to maintain constant characteristics through successive generations only when all parts of the group are equally fertile. If the fertility of one group or class exhibits a tendency to increase, the group in time tends to impress its characteristics to a greater degree upon the whole group. If, for any reason the opposite condition prevails, the part loses in influence.

When applied to human society the tendency can be readily seen. If an increased birth rate occurs in the upper classes with or without a decrease in the lower strata, the prevailing characters are higher. If for any reason the lower classes tend to multiply more rapidly than the upper, the social tendency is downward. When a decreasing birth rate occurs in the class most noted for its stability there arises a cause for serious consideration. France has for a generation or more faced a declining birth rate. Germany is now facing the same condition. England, during the last decade, has noted a decline. Even in this country it is perceptible. In France the number of marriages have not decreased. It is apparent that the size of the family is growing smaller. Whenever the size of the family declines below a certain point (four children) the population ceases to grow, remains stationary, or may fall off. If a decline in fertility occurs, it is matter of grave concern in what strata it occurs. If evenly in all classes, then no change in society is made; but if unevenly, society must change to suit the ideals of the predominating group.

The source of the population is always a matter of concern. Pearson points out that 25 per cent of the married couples produce 50 per cent of the children. These children make the future citizens. It is therefore of the utmost concern whence this per cent comes. A study of the relative size of families in several countries may throw some light on this subject and illustrate our contention. It is a well known fact that high fertility and high desirability are not always found in the same class. This is clearly illustrated by two classes of American society. Among American graduates the average number of children in the family is two. This represents the most desirable of American citizenship in which the number of children falls below the number necessary to maintain itself. Among American deaf-mutes, a highly undesirable class, the average number of children per family is 6.1. In England the contrast is more marked. Among English intellectuals the number of offspring per marriage is 1.5, while among the English deaf-mutes the number rises to 6.2.

In order for a people to maintain its normal population, each marriage must result in the production of four children. If the population is growing the number of offspring will exceed this number. According to Pearson, the family records of normal individuals show an average of 5.3 children. The same is true of the Danish working people, and the Australian working class. The intellectual classes fall below this number, while the defective classes rise above it. In the city of London the mentally defective average 7 children, while the criminal class average 6.6 per family. According to these statistics the highest fertility exists among the most undesirable. The marked feature is the facility with which the intellectual class eliminates itself. An illustration of this may be seen in the relative proportion of whites and blacks in the Islands, lying between the Gulf of Mexico and the Carribean Sea. The early settlers carried their slaves with them that they might live in ease. The blacks today vastly outnumber the whites. But one consequence can follow if this ratio persists—like the blacks of Barbadoes, the offspring of the defective classes will greatly outnumber the offspring of the normal. The intellectuals, in order to maintain their status, must follow the conclusion of the Frenchman: "My father was a farmer, I am a professor, my son must be a farmer."

A study of communities gives some interesting conclusions. If we take those communities in which the larger number of professional men reside, we find always the lower birth rate. The same condition prevails in those communities in which a large number of domestics are employed. In other words, these conditions which should make for the greater comfort in child bearing and child rearing operate to decrease the birth rate. High social status plainly operates to reduce fertility. On the other hand those communities in which are to be found many pawn shops, child laborers, defectives, over crowding, etc., there is a correspondingly higher birth rate.

This gives us an idea whither society tends. Here again facts come to our support. Sixty years ago in London this condition was reversed. In 1851 the higher the number of professional men and well-to-do families in a community, the higher the birth rate, and the higher the number of undesirables in a district, the lower the birth rate. This change is going on in every civilized land and it is not without a positive significance.

In the ten years preceding 1901 the number of children in London declined over 5000, notwithstanding that the population increased 300,000 in the same time. This falling off occurred among the better classes, among the thrifty and the independent.

There is another respect in which the size of the family acts for or against maintaining the social status. A careful study of the incidence of disease, insanity, criminality, etc., among the children of families in reference to the relative frequency of those conditions among the first, second and third born shows that the first child is twice as apt to suffer from chronic disease as the third, the second born being between these two. The same thing is true in regard to mental defects. Insanity occurs much more frequently among the first born than among either the second or third born. The first born again are about three times as apt to be criminal as the third born. These conclusions are based on the study of a large number of families. Note the effect of the small family upon the status of society. The small family contributes on the average much more than its share of the diseased, the defective, and the criminal classes.

We recall the proposition that a group of organisms will tend to maintain constant characteristics through successive generations only when all the parts of the group are equally fertile. Human society follows the same law. It is plainly evident that in all civilized lands at the present moment the lower strata of society are much more prolific than the higher. It may, therefore, take but a few generations to carry the type which a generation ago was of the higher strata of society to the lower strata with all its burdens of mental, physical and moral deficiency.

In the earlier stages of the human race natural selection could be depended upon to eliminate a certain proportion of the unfit. But we have advanced far into a period where the survival of the fittest is supplanted by the survival of a large class which owes its very existence to the advance in sanitary science. Therefore the balance that was maintained by the more virile members is destroyed. Our social instincts lead us to protect the weak. This very protection brings consequences against which society must now seek to protect itself.

Artificially we divide society into three great classes, the professional, the middle, and the working class. To the first belong those engaged in highly intellectual pursuits; to the second belong the better class of farmers, merchants and skilled laborers, and to the third the great army of unskilled labor. This middle class forms the most stable portion of society. From it come the thinkers—the real brains of the nation. Relatively this class produces the far greater proportion of ability. We sometimes make the mistake of assuming that we can educate ability into the people. Ability is inherited—and no amount of education can replace brains. The difference between the middle class and the lower is not education, it is stock. Commercially, this idea is well understood and taken advantage of. It is therefore a matter of concern when we discover a reversal of conditions of fifty years ago. We actually find today a growing decline in the birth rate among the upper classes. This symptom of the social malady calls for serious thought. Greece and Rome fell from lack of stock. Other nations that heed not the lessons taught by history are in a fair way to attain the same end. We express horror at human warfare, but many of the practices of society are more disastrous in ultimate analysis than any war can ever be.

We do not need to look very sharply to see this process of the elimination of the higher classes going on in our own country. A recent writer in speaking of "childless Americans," said the "good old American stock" will soon be a thing of the past. Many others have noticed this gradual displacement of the American family. The writer counted up some sixty-five families, all New England stock, and found one hundred and fifteen children—less than two per family. The number of children born to these parents, providing each child lives to maturity, will not replace the parents.

Why do American families have so few children? The high cost of living has been assigned as a cause. It probably is a factor to some extent. But the poorer people have several children in each family. You may see the childless mansion, and the hovel overflowing with children side by side. Our grand parents reared families of eight to ten children, our parents families of five to eight, and we feel that we have done our duty with only one or two children to offer as our contribution to the future. Our grandfathers had no bank accounts, our fathers did better in this respect, and we better still. Yet the family steadily diminishes.

A study of these American families of the better-to-do class reveals several causes. Later marriages play a part. Our grandmothers married at twenty, our mothers at twenty-two, and ourselves at twenty-five or later. Young people are prone to imitate the example of others, and for the first years desire a good time. This class in time comes to look upon parenthood as an unfortunate accident. Social duties crowd upon them, and they soon forget the duty of parenthood. While many causes may be operative in reducing the birth rate among this class, the chief cause is undoubtedly willful selfishness. In this way the old American stock is eliminating itself from the equation of maintaining the social status in the very land it sought to establish for itself and its children forever, deliberately and with malice aforethought.

To the student of sociology these symptoms are significant. They point to a want of racial stability. The great problems of the near future are sociologic problems. Upon their solution will depend the upward evolution of the people. There will be evolution, but if present conditions persist, it must be toward lowered ideals.

No prophet has yet arisen to point the way, but the first step must be in the direction of a more equal distribution of wealth. The present condition, in a racial sense, is harmful to both those that have and those that have not. Some form of legislation looking to the limitation both of the size of individual fortunes and the minimum of wages paid labor must be enacted. The power of the ultra rich must be lessened, and the compensation of the wage earner raised to the living point, where the child of the laborer has a fair chance in the competitive struggle for success. Business must be content with reasonable service and reasonable profits.

The existence of disease, both in adult and infant life, should be treated as a national problem. There is no more excuse for the three or four hundred thousand cases of typhoid fever each year than there would be for that many cases of smallpox. The cost of national, State and municipal neglect to enforce sanitary measures is assessed at half a million lives annually. We are all agreed that the high infant mortality prevailing in this country is not the working out of nature's law of the survival of the fittest. Yet we permit one-fourth of a million of such to die each year. With all that has been written on the subject of tuberculosis in the last ten years, we are a long way from possessing definite statistics of its prevalence. It is said that a Turkish official when asked about the death rate in Turkey, replied: "Allah wills that all shall die; some die young and some die old; but Allah wills that all shall die." This is about as far as our accurate knowledge goes. Sporadic campaigns have been undertaken here and there, but a national campaign is needed. Weak municipalities and weak States need to be spurred into respectability for the general good.

The annual crop of feeble-minded children could be reduced very materially by a little wise legislation. New York State has 30,000 such unfortunates, only one-fifth of whom are in State institutions. It costs the State $5000 to rear a feeble-minded child. Is it economy, is it common sense, is it justice, that this class should be at large free to beget their kind?

The number of insane may be overestimated, the rate of increase of insanity may not be so great as reported, but it is bad enough to justify a national commission. It might represent sounder business to spend a few millions in the study of human conservation than other millions in the study of tariffs and trusts. To relieve the burden of dependency might do more to reduce the cost of living.

The business man studies the source of his income. It would seem a wiser policy for organized government to study the source of its future citizenship, to correct the present evils associated with marriage and to exact equal justice for all.

---

Subacromialbursitis resulting from indirect violence (the usual cause, is often, if not always, associated with and due to injury to the supraspinatus tendon. A calcareous deposit often forms in the tendon which in the x-ray plate must be distinguished from fracture of the greater tuberosity.—*American Journal of Surgery.*

